# Predicting asthma using imbalanced data modeling techniques: Evidence from 2019 Michigan BRFSS data

**DOI:** 10.1371/journal.pone.0295427

**Published:** 2023-12-07

**Authors:** Nirajan Budhathoki, Ramesh Bhandari, Suraj Bashyal, Carl Lee

**Affiliations:** 1 Department of Statistics, Actuarial & Data Sciences, Central Michigan University, Mount Pleasant, Michigan, United States of America; 2 Department of Physics, Central Michigan University, Mount Pleasant, Michigan, United States of America; 3 Department of Geography & Environmental Studies, Central Michigan University, Mount Pleasant, Michigan, United States of America; University of Ghana, GHANA

## Abstract

Studies in the past have examined asthma prevalence and the associated risk factors in the United States using data from national surveys. However, the findings of these studies may not be relevant to specific states because of the different environmental and socioeconomic factors that vary across regions. The 2019 Behavioral Risk Factor Surveillance System (BRFSS) showed that Michigan had higher asthma prevalence rates than the national average. In this regard, we employ various modern machine learning techniques to predict asthma and identify risk factors associated with asthma among Michigan adults using the 2019 BRFSS data. After data cleaning, a sample of 10,337 individuals was selected for analysis, out of which 1,118 individuals (10.8%) reported having asthma during the survey period. Typical machine learning techniques often perform poorly due to imbalanced data issues. To address this challenge, we employed two synthetic data generation techniques, namely the Random Over-Sampling Examples (ROSE) and Synthetic Minority Over-Sampling Technique (SMOTE) and compared their performances. The overall performance of machine learning algorithms was improved using both methods, with ROSE performing better than SMOTE. Among the ROSE-adjusted models, we found that logistic regression, partial least squares, gradient boosting, LASSO, and elastic net had comparable performance, with sensitivity at around 50% and area under the curve (AUC) at around 63%. Due to ease of interpretability, logistic regression is chosen for further exploration of risk factors. Presence of chronic obstructive pulmonary disease, lower income, female sex, financial barrier to see a doctor due to cost, taken flu shot/spray in the past 12 months, 18–24 age group, Black, non-Hispanic group, and presence of diabetes are identified as asthma risk factors. This study demonstrates the potentiality of machine learning coupled with imbalanced data modeling approaches for predicting asthma from a large survey dataset. We conclude that the findings could guide early screening of at-risk asthma patients and designing appropriate interventions to improve care practices.

## Introduction

In general terms, the Centers for Disease Control and Prevention (CDC) defines asthma as a disease that affects lungs. It is one of the most common long-term diseases in children, but it can also affect adults. CDC identifies that asthma causes repeated episodes of wheezing, breathlessness, chest tightness, and nighttime or early morning coughing [1]. Both incidence and prevalence of asthma have been increasing in the United States: current asthma prevalence increased from 7.2% in 2001 to 9.0% in 2019 [2]. Michigan has been observed to have higher asthma prevalence rates than the national average, with an estimated 11.1% adults having current asthma based on the 2019 Behavioral Risk Factor System Surveillance (BRFSS) data [2]. While there is no known cure for asthma, it is possible to control the condition by avoiding things that trigger asthma attacks and receiving adequate medical attention [1].

Machine learning algorithms have been extensively used for predicting health outcomes such as diabetes [3], breast cancer [4], and coronary artery disease [5] among others. Prior studies on asthma typically focused only on studying risk factors using traditional statistical modeling methods such as logistic regression [6–8]. Moreover, studies that used modern machine learning methods mainly relied on clinical factors extracted from electronic health records or during home telemonitoring [9, 10].

Regarding the use of imbalanced data modeling techniques in machine learning, Alghamdi et al. [11] developed models to predict incident diabetes from an imbalanced data that was treated using the Synthetic Minority Over-Sampling technique (SMOTE). Another study developed models for sleep/awake classifications from imbalanced Fitbit data [12]. The study compared the performances of four resampling strategies, namely random up sampling, random down sampling, Random Over-Sampling Examples (ROSE), and SMOTE, and found that ROSE consistently outperformed other methods. Both studies demonstrated that a balanced dataset produces better predictive performance of machine learning models.

We did not find studies that evaluated machine learning performances in asthma prediction using demographic/socioeconomic, personal habits, and health characteristics collected during national surveys as predictors. Moreover, imbalanced data issues were not studied in the context of survey data in the cited literatures.

This study aims to achieve two main objectives: (i) To assess and compare the effectiveness of machine learning (ML) models in predicting asthma among Michigan adult population and (ii) To select an optimal model for further investigation into the risk factors. Our study is unique in that it first addresses the imbalanced data issue in a large-scale national survey and then uses ML algorithms for asthma prediction. The order of variable importance for making predictions in each algorithm is also presented. Furthermore, we thoroughly examine the “best” model to study the association between the outcome variable “ASTHMA” and the predictors considered in the study.

## Materials and methods

### Data

Data for this study is taken from the 2019 BRFSS conducted among Michigan adults. BRFSS is an annual telephone survey, incorporating both landlines and cellular phone users, managed by state health departments in all US states and territories with support from CDC. Study participants are chosen based on a random sampling method. BRFSS seeks to collect data on health-related risk behaviors, chronic health conditions, healthcare access, and use of preventive services from the noninstitutionalized adult population (≥18 years) residing in the United States. BRFSS data and documentation are publicly available on the CDC website [13].

### Outcome variable and predictors

The outcome variable for this study is whether a survey participant has self-reported current asthma or not. BRFSS constructs two different asthma measures: Lifetime Asthma and Current Asthma. In the survey, lifetime asthma is defined as an affirmative response to the question, “Have you ever been told by a doctor (nurse or other health professional) that you have asthma?” Current asthma is defined as an affirmative response to that question followed by an affirmative response to the subsequent question, “Do you still have asthma?” [2]

Predictor variables for this study were chosen based on relevant literature reviews [6–8, 14–17]. Table 1 presents a categorization and description of the variables used in this study.

**Table 1:** Variable description.

Variable Type	Variable names used in BRFSS (Labels)
Demographic/Socioeconomic Characteristics (DS)	SEX (Sex), AGE_G (Age group), EDUCAG (Education), INCOMG (Income), IMPRACE (Race), URBSTAT (Residence), VETERAN3 (Veteran Status), EMPLOY1 (Employment), MEDCOST (Could not see doctor due to cost)
Personal Habits (PH)	SMOKER3 (Smoker status), USENOW3 (Smokeless tobacco products), SMOKE100 (Smoked at least 100 cigarettes)
Health Characteristics (HC)	DIABETE4 (Diabetes), BMI5CAT (Body Mass Index), CHCCOPD2 (Chronic Obstructive Pulmonary Disease), FLUSHOT7 (Flu shot/spray past 12 months)

### Data preprocessing

Frequently, statistical analysis cannot be directly applied to survey data without performing proper data cleaning. We particularly checked data for missing values as their occurrences are common in survey data. The outcome variable as well as some predictor variables contained missing values that ranged from 0.15% for the variable *Diabetes* to 6.24% for the variable *Body Mass Index*. Whenever a missing value was present in the outcome variable, we removed the entire row from the dataset. Even after removing the missing data in the outcome variable, there were still some missing data in the predictors. We performed imputation using *mice* package [18] in R [19] for the missing data in the predictors. This package uses imputation method involving chained equations, where each imputation of a missing value is informed by the imputed values of other variables. Although we did not examine the missing data mechanism in this study, it is known that *mice* can handle data that are Missing at Random (MAR) or Missing Not at Random (MNAR).

### Machine learning models

The machine learning models used in this study are briefly summarized in the following.

#### Logistic regression (LR)

This is a parametric modeling technique and has a major advantage of being easily interpreted. The technique is widely used for categorical target, especially for binary target (Y). Denoting set of predictors by X, the probability that Y takes a value of 1 is computed using logistic function,

$$p\left(Y=1\right)=\frac{e^{\beta_{0}+\beta_{1}X}}{1+\;e^{\beta_{0}+\beta_{1}X}}$$

With some manipulation,

$$\text{log}\;\left(\frac{p\left(Y=1\right)}{1-p\left(Y=1\right)}\right)=\beta_{0}+\beta_{1}X$$

The left-hand side is the log-odds or logit [20]. The exponentiation of logit gives odds ratio which indicate the odds of an event occurring in one group compared to the odds of it occurring in another group.

#### Partial least squares (PLS)

This technique reduces the predictors to a smaller set of uncorrelated components and performs least squares regression on these components rather than on the original predictors. PLS regression is especially useful when predictors are highly collinear, or when there are more predictors than observations. The technique reduces the number of predictors using a technique similar to principal components analysis to extract a set of components that describes maximum correlation between the predictors and response variables [20, 21]. Let Y, *n* x *q* matrix, be the response variables and X, *n* x *p* matrix, be a set of predictors. If Y is a univariate response variable, then *q* = 1. Assuming X and Y are standardized, then PLS method attempts to determine a linear decomposition of X and Y as X = TP’ and Y = UQ’, where T and U are both *n* x *r* matrix. The T matrix is the X-principal component. Each X-principal component is a linear combination of X, and U is the Y-principal component. Then, regression is performed between T, as the predictors and U as the response variables.

#### Random forest (RF)

This technique involves building several decision trees on bootstrapped training samples [20]. When trees in random forest are built, only a random sample of ‘m’ predictors is chosen as split candidates from the full set of ‘p’ predictors in each split. By allowing only a subset of predictor at each split, the correlation between trees will be reduced which in turn makes the average of resulting trees less variable. When many correlated predictors are present, a small value of ‘m’ is desirable. This ‘m’, known as tuning parameter for random forest algorithm is tuned to get optimal model.

#### Gradient boosting (GB)

Random Forest constructs multiple trees on bootstrapped datasets, resulting in independently grown trees. With boosting, each tree is grown sequentially using the information from previously grown trees. The technique starts with fitting a small tree, often with two or four terminal nodes. Given the current model, another small decision tree is fitted to the residual from the model. The new tree is added to the fitted function, and residual is recomputed. The fitted function is slowly improved until the improvement is ignorable. This method has three tuning parameters: the number of trees, shrinkage parameter that controls the rate at which boosting learns, and number of splits in each tree [20]. Grid method and cross-validation are often applied to achieve the final best model.

#### Least absolute shrinkage and selection operator (LASSO)

This is known as a shrinkage method that shrinks the coefficient estimates towards zero while fitting a model. While the least squares method minimizes the residual sum of squares (RSS) given by $\sum_{i=1}^{n}\left(y_{i}-\beta_{0}-\sum_{j=1}^{p}\beta_{j}x_{ij}\right)$, LASSO works by minimizing the quantity $\sum_{i=1}^{n}\left(y_{i}-\beta_{0}-\sum_{j=1}^{p}\beta_{j}x_{ij}\right)+\;\lambda \sum_{j=1}^{p}\left|\beta_{j}\right|$, where the second term is called a shrinkage penalty [20, 22]. The tuning parameter λ controls the relative impact of the two terms viz. RSS and shrinkage penalty on the regression coefficient estimates. The value of λ is often chosen by cross-validation method. LASSO produces simpler and more interpretable models that involve only a subset of predictors since some of the estimates shrink to zero at the price of potential increase in model bias.

#### Elastic net (EN)

This method seeks to minimize a different quantity compared to LASSO which is given by $RSS+\;\lambda \;\sum_{j=1}^{p}\beta_{j}^{2}+(1-\lambda )\sum_{j=1}^{p}\left|\beta_{j}\right|$ [22]. It is a weighted combination of LASSO and ridge regression. Elastic net controls the impact of correlated predictors. The two tuning parameters: λ for complexity and α for the compromise between LASSO and ridge are tuned to obtain the optimal model.

#### K-nearest neighbors (KNN)

It is a simple, easy-to-implement, non-parametric supervised machine learning algorithm. Each predicted response at a given predictor is the average of the responses at the K nearest predictors. The choice of K, which is the tuning parameter for this method, decides the smoothness of the predicted response surface [20]. The curse of dimensionality quickly occurs when the number of predictors increases.

#### Support vector machine (SVM)

SVM was originally developed for classification problems but is also used in regression. The idea behind SVM is to seek for a hyperplane that differentiates the classes as accurate as possible [20]. Consider a binary classification problem. If the two classes are separatable by the predictors, there exists many possible hyperplanes to separate the two classes. SVM seeks to identify the hyperplane that has maximum margin for separating the two classes as the classifier. If the two classes are not separatable by the predictors, there are points that will be wrongly classified. A slack variable is introduced and SVM seeks to identify the hyperplane that has the maximum margin as the classifier. The hyperplane may be linear or non-linear. For problems involving non-linear hyperplane, a kernel function is introduced to capture the non-linear characteristics.

### Statistical analysis

A preliminary screening of the predictors to be included in the machine learning models was conducted using the Chi-Squared association test. We present the frequencies for raw data and the corresponding weighted percentages of the distribution of participants in various levels of the predictors. These weighted estimates, obtained using R *survey* [23] package with CDC-computed study-specific weights provided for each respondent, are generalizable to the entire Michigan population. Predictors with p-value < 0.05 were considered statistically significant and were selected for model building. We also present weighted odds ratio and their corresponding 95% confidence interval (CI) for further exploration of risk factors in the logistic regression model. All analyses were conducted using R.

### Dealing with imbalanced dataset

Data from 10,337 survey participants were considered for analysis in this study. Of those, 10.8% were told by doctor (nurse or other health professional) that they had asthma at some period before the survey and were still having it. The majority (89.2%) of the survey participants reported not having asthma. This is indicative of an imbalanced class problem in the context of machine learning applications. The result of this disparity in distribution of the outcome variable is that the predictive algorithms perform poorly as we present later in Table 4.

Various techniques including resampling and cost-sensitive learning have been developed that attempt to address class imbalance before building predictive models [24, 25]. The resampling techniques that involve generating synthetic data to increase minority class instances during the pre-modeling stage are among the popular ones. Examples of these techniques are ROSE [26] and SMOTE [27]. ROSE generates synthetic data for the minority class based on a smoothed bootstrap approach and simultaneously the majority class is under sampled without replacement to obtain a more balanced data. SMOTE generates new samples of the minority class using the nearest neighbors of these cases, and the majority class is under sampled to obtain a more balanced dataset. The choice of sample size and the proportion of each class for the adjusted data set are specified by the user. In this study, we choose that the adjusted data is balanced at 50% for each class and the sample size remains the same as the original training data set. Details on these two techniques can be found in [26, 27] and the references therein. The comparison study of different synthetic data generating techniques for imbalanced data classification in [12] indicated ROSE performed the best in different applications. In this study, we employ both ROSE and SMOTE techniques during the pre-modeling data processing stage. We then build predictive models from the balanced training sets and compare performance on still-imbalanced test sets.

The ROSE method has been applied using the R package ROSE [26] with over-sampling to generate synthetic data for minority class (ASTHMA = “YES”) and under sampling to select majority class (ASTHMA = “NO”) so that proportion of both classes is approximately 50% in the resulting dataset. For SMOTE implementation, we use the R package DMwR [28] with the default k value of 5 for the embedded K-nearest neighbor technique. Again, the majority and minority classes were adjusted such that they become approximately of the same size. Table 2 summarizes the distributions of original training data, after-ROSE, and after-SMOTE training data in the two asthma classes.

**Table 2:** Distribution of outcome variable “ASTHMA” in the original, after-ROSE, and after-SMOTE training data.

	N	ASTHMA = “YES”	ASTHMA = “NO”	Proportion of “YES”
Original training data	7235	782	6453	10.8%
After-ROSE training data	7235	3597	3638	49.7%
After-SMOTE training data	7235	3910	3753	51.0%

### Model building, validation and evaluation

The entire dataset was partitioned into training data (70%) and testing data (30%). The training set was used to build and select the “best” model within each modeling technique based on results of 10-fold cross-validation for parameter tuning. All machine learning models were built using *caret* package (short for **C**lassification **A**nd **RE**gression **T**raining) [29] in R. Once the optimal models for each technique were obtained, test data were used for evaluating model performances. It is to be noted that the test dataset remained imbalanced (N = 3102, N (ASTHMA = “YES”) = 336 and N (ASTHMA = “NO”) = 2766); therefore, we use performance metrices such as F-measure, G-measure, Matthews Correlation Coefficient, and Cohen’s Kappa that are common for evaluating performances in the presence of imbalanced classes [30]. We also reported sensitivity, accuracy, and area under the receiver operating characteristics (ROC) curve values to evaluate model performances [31]. For each of these measures, a higher value denotes better predictive ability of the models. Additionally, we tabulated variable importance in each of the machine learning models obtained using standard method in the *caret* package. This method works by using the model to predict outcome on the test data and then examining the difference in prediction accuracy with and without each predictor variable.

## Results

The distribution of study participants in various levels of the predictors as well as results for the Chi-squared test of association is presented in Table 3.

**Table 3:** Association between the outcome variable “ASTHMA” and predictor variables in the study (n = 10337).

Predictors	ASTHMA			
	No (N = 9219)	Yes(N = 1118)	Chi-Square	P-value
	N (Weighted %)	N (Weighted %)		
**Sex**				
Female	4978 (86.5)	755 (13.5)	62.653	< 0.01
Male	4241 (91.4)	363 (8.6)		
**Age group**				
18–24	634(87.1)	91(12.9)	27.033	0.02
25–34	953(89.0)	120(11.0)		
35–44	986(88.1)	126(11.9)		
45–54	1315(87.8)	195(12.2)		
55–64	1800(88.1)	223(11.9)		
65 or older	3531(91.7)	363(9.3)		
**Education**				
Below High School	342(82.6)	66(17.4)	45.834	< 0.01
High School	2372(89.5)	303(10.5)		
Some college or more	6505(89.6)	749(10.4)		
**Income**				
Below $25000	1586(82.3)	323(17.7)	123.52	< 0.01
$25000–$50000	1881(88.6)	233(11.4)		
$50000 or more	4141(91.6)	393(8.4)		
Don’t know/Not Sure	1611(90.0)	169(10.0)		
**Race**				
White, Non-Hispanic	7751(89.5)	897(10.57	27.206	< 0.01
Black, Non-Hispanic	736(84.5)	123(15.51		
Asian, Non-Hispanic	142(93.0)	7(7.08		
Hispanic	242(89.9)	28(10.1)		
Other	348(86.5)	63(13.5)		
**Residence**				
Urban counties	8477(88.9)	1029(11.1)	0.0030	0.97
Rural counties	742(88.8)	89(11.2)		
**Veteran Status**				
Yes	1061(92.7)	85(7.3)	16.90	< 0.01
No	8158(88.5)	1033(11.5)		
**Employment**				
Employed	4427(89.8)	479(10.2)	131.78	< 0.01
Out of work	387(87.6)	56(12.4)		
Homemaker	441(87.1)	54(12.9)		
Student	286(89.6)	39(10.4)		
Retired	3108(91.3)	315(8.7)		
Unable to work	570(77.6)	175(22.4)		
**Smoker Status**				
Current smoker	1355(85.4)	226(14.6)	29.504	< 0.01
Former smoker	2759(89.4)	314(10.6)		
Never smoked	5105(89.8)	578(10.2)		
**Smokeless tobacco products**				
Yes	266(84.0)	32(16.0)	9.8302	0.06
No	8953(89.1)	1086(10.9)		
**Smoked at least 100 cigarettes**				
Yes	4106(87.7)	541(12.3)	12.073	0.01
No	5113(89.9)	577(10.1)		
**Diabetes**				
Yes	1189(83.9)	206(16.1)	33.366	<0.01
No	7846(89.6)	878(10.4)		
Pre-diabetes or borderline diabetes	185(85.0)	34(15.0)		
**Body Mass Index**				
Underweight	132(85.4)	20(14.6)	74.32	<0.01
Normal Weight	2648(91.1)	244(8.9)		
Overweight	3328(90.9)	335(9.1)		
Obese	3111(85.5)	519(14.5)		
**Chronic Obstructive Pulmonary Disease**				
Yes	689(67.8)	317(32.2)	422.73	<0.01
No	8530(90.8)	801(9.2)		
**Could not see doctor due to cost**				
Yes	839(83.3)	161(16.7)	43.619	<0.01
No	8380(89.7)	957(10.3)		
**Flu shot/spray past 12 months**				
Yes	4363(87.7)	585(12.3)	10.652	0.02
No	4856(89.7)	533(10.3)		

Counts provided (N) are for raw data. The weighted %, Chi-square test statistics, and their corresponding p-value are obtained using R survey package with CDC-computed study-specific weights provided for each respondent.

As Table 3 shows, asthma is more prevalent among females than males (13.5% vs 8.6%). The age-group of 45–54 years had the highest asthma prevalence, while 65 years or older had the lowest prevalence. Both the predictors sex and age-group had statistically significant association with asthma. Education was also found to have significant association with asthma as those below high school was the most prevalent group. Regarding income, participants in the lowest income group (below $25000) were the ones who reported asthma more than other groups. Black, Non-Hispanic communities reported higher asthma rates than White, Non-Hispanic, Asian, and Hispanic group. Again, both the predictors income and race seemed to have significant association with asthma.

No significant association was found between urban/rural residence and asthma. Regarding veteran status, those who did not report of being a veteran had higher asthma rates (11.5% vs 7.3%). Higher asthma rates were also observed among those who were unable to work, current smokers, and smoked at least 100 cigarettes in their life. Diabetes status was also found to be significantly associated with asthma as more people having diabetes or borderline diabetes had asthma compared to people without diabetes. Underweight, overweight, and obese categories had higher asthma rates than reported by people in normal weight categories. There seems to exist a strong association between chronic obstructive pulmonary disease (COPD) and asthma since nearly one-third of the people with the disease had asthma, while only 9.2% of the people without the disease had asthma. Also, higher asthma rates were reported by people who could not see a doctor due to the medical cost involved (16.7% vs 10.3%). The study also showed that people who received a flu shot/spray in the past 12 months had slightly higher asthma rates than those who did not receive the shot (12.3% vs 10.3%).

To summarize, asthma was found to have a statistically significant association with demographic/socio-economic variables namely sex, age-group, education, income, race, veteran status, employment, and inability to see doctor due to cost. Personal habits, namely smoker status and smoked at least 100 cigarettes throughout the life were also significantly associated with asthma. Regarding health characteristics, diabetes, body mass index, COPD, and flu shot/spray during the past 12 months were found to have a significant association with asthma. The variables with significant association were chosen as predictors to build predictive models.

We compared the model performances between modeling based on the original imbalanced training data and both the ROSE-adjusted as well as SMOTE-adjusted balanced training data. Table 4 summarizes the performances of models trained on the original imbalanced data.

**Table 4:** Performance of machine learning models on test set using the original imbalanced training set.

Technique	Sensitivity	Accuracy	Area under ROC curve	F Measure	G Measure	Matthew’s Correlation Coefficient	Cohen’s Kappa
Logistic Regression	0.054	0.895	0.525	0.099	0.231	0.168	0.084
Partial Least Squares	0.006	0.892	0.503	0.012	0.077	0.056	0.010
Random Forest	0.000	0.892	0.500	0.000	0.000	NaN*	0.000
Gradient Boosting	0.003	0.892	0.501	0.006	0.055	0.032	0.005
LASSO	0.042	0.894	0.520	0.079	0.204	0.153	0.067
Elastic Net	0.021	0.893	0.510	0.040	0.144	0.108	0.034
KNN	0.009	0.893	0.505	0.018	0.094	0.089	0.016
SVM (Linear Kernel)	0.000	0.892	0.500	0.000	0.000	NaN	0.000

*Denotes Not a Number

With very small sensitivity values, results in Table 4 indicate that the predictive models had poor performance in predicting asthma cases (ASTHMA = “YES”). Accuracy measures appear to be quite high, while the fitted model is very poor, if not totally useless. Higher accuracies result due to models being strongly biased towards larger proportion of majority class, so that does not effectively provide useful statistic for classification of minority class. Moreover, area under ROC curve values are barely over 0.5, which suggests these modeling techniques are not adequate for modeling the original imbalanced asthma data.

Potential problems that may cause the weak performances of these modeling techniques may include:

(a) The predictor variables surveyed in the BRFSS may not be appropriate for predicting asthma. Some additional predictor variables may need to be considered.
(b) Additional data cleaning and manipulations may provide better prediction of asthma.
(c) Other modeling techniques may be more appropriate.
(d) Our dataset contains only 10.8% of ASTHMA = “YES” cases. This is an imbalanced data issue. The typical modeling techniques require the assumption that the data is approximately balanced. One cannot apply modern machine learning techniques to classify imbalanced data effectively.

Regarding (a), BRFSS is a national survey that relies on self-reported data by the survey participants during telephonic interviews. Unlike other national surveys such as the National Health and Nutrition Examination Survey (NHANES) [32], no laboratory tests are performed which may provide better measures of individual health conditions. For BRFSS dataset, we reviewed the variables used in similar studies in the literature and concluded that the predictor variables used are solid and appropriate for our study. However, there may be additional variables, such as family history of asthma, that could help asthma prediction, but were not collected in the survey. Regarding (b), possible additional data manipulation and variable transformations may be performed by using different numerical transformations. We reviewed various related literatures and chose a similar approach by keeping the data in their original scales, so that the resulting selected predictors can be meaningfully interpreted. Regarding (c), the eight modeling techniques applied are the common modern machine learning techniques, which have shown evidence of their successes in many applications. We did not include the modern deep learning techniques because the observational survey data has a weak signal-to-noise ratio, which is the major weakness of deep learning techniques. In addition, we would like to be able to observe predictors that show a high association relationship with asthma. Deep learning cannot provide such information. Thus, our attention is directed towards (d) to address the issue caused by imbalanced classes in terms of the outcome variable. As described in the Materials and Methods section, the ROSE method was implemented to create a balanced training dataset, and the model performance in the test dataset is presented in Table 5.

**Table 5:** Performance of machine learning models on test set using the ROSE-adjusted balanced training set.

Technique	Sensitivity	Accuracy	Area under ROC curve	F measure	G measure	Matthew’s Correlation Coefficient	Cohen’s Kappa
Logistic Regression	0.500	0.716	0.621	0.276	0.609	0.167	0.142
Partial Least Squares	0.494	0.734	0.629	0.287	0.614	0.181	0.157
Random Forest	0.307	0.739	0.549	0.203	0.492	0.073	0.067
Gradient Boosting	0.500	0.6911	0.624	0.280	0.612	0.172	0.147
LASSO	0.506	0.6847	0.628	0.285	0.616	0.178	0.153
Elastic Net	0.506	0.6837	0.628	0.284	0.616	0.177	0.151
KNN	0.536	0.624	0.586	0.236	0.583	0.109	0.081
SVM (Linear Kernel)	0.417	0.751	0.604	0.266	0.574	0.154	0.139

Among the models developed, five had similar performances in terms of the metrics considered. These were logistic regression, partial least squares, gradient boosting, LASSO, and elastic net, with sensitivity at around 50% and area under the curve (AUC) at around 63%. For comparison purpose, we also present the result in Table 6, which were obtained using SMOTE to create a balanced training dataset and then evaluated on the imbalanced test dataset.

**Table 6:** Performance of machine learning models on test set using the SMOTE-adjusted balanced training set.

Technique	Sensitivity	Accuracy	Area under ROC curve	F measure	G measure	Matthew’s Correlation Coefficient	Cohen’s Kappa
Logistic Regression	0.443	0.746	0.613	0.274	0.589	0.164	0.147
Partial Least Squares	0.429	0.757	0.613	0.276	0.584	0.167	0.152
Random Forest	0.420	0.722	0.589	0.246	0.564	0.126	0.110
Gradient Boosting	0.354	0.791	0.599	0.268	0.547	0.161	0.154
LASSO	0.432	0.753	0.612	0.275	0.585	0.165	0.149
Random Forest	0.420	0.722	0.589	0.246	0.564	0.126	0.110
Gradient Boosting	0.354	0.791	0.599	0.268	0.547	0.161	0.154
LASSO	0.432	0.753	0.612	0.275	0.585	0.165	0.149
Elastic Net	0.432	0.753	0.612	0.274	0.585	0.164	0.149
KNN	0.244	0.842	0.579	0.250	0.472	0.162	0.162
SVM (Linear Kernel)	0.411	0.746	0.599	0.260	0.569	0.145	0.131

A comparison of the results in Table 4 versus Tables 5 and 6 suggests that the performance of the models that were trained using the synthetic data is better than those trained using the original data. For top performing models built using ROSE-adjusted training set, the area under the ROC curve values rose to around 0.63 compared to the values near to 0.5 on the original imbalanced training set. Similarly, for the top performing models built using SMOTE-adjusted training set, the area under the ROC curve values were found to be close to 0.61. Although accuracy values have been reduced compared to those in Table 4, they now give a true picture of models’ abilities in classifying both the majority and minority classes. Therefore, this result demonstrates the improved potentialities of machine learning models that have been trained on a dataset balanced using both ROSE and SMOTE methods. Considering the higher importance of models’ ability to correctly classify asthma cases, as reflected by sensitivity values, one may observe that ROSE outperforms SMOTE in this study.

The order of variable importance for each modeling technique is shown in Table 7. These results, obtained from the ROSE-adjusted models, suggest that the predictor *Chronic Obstructive Pulmonary Disease* is the most important for making prediction in each model. This is in line with the result in Table 1 where we presented that almost one-third of the participants with *COPD* had asthma. The next is the predictor, *Income*, followed by *Sex* and *Employment*. However, the degrees of importance of predictors across models are not consistent, except for *COPD*. Both smoking related predictors do not seem to have a high contribution in predicting asthma when taking other predictors into the model building.

**Table 7:** Variable importance order from ROSE-adjusted balanced training set.

Factors Selected	Models								Average rate
	LR	PLS	RF	GB	LASSO	EN	KNN	SVM	
Chronic Obstructive Pulmonary Disease	1*	1	1	1	2	2	1	1	1.25
Income	4	4	3	9	5	5	2	2	4.25
Sex	2	3	5	3	7	7	5	5	4.625
Employment	6	5	7	6	4	4	4	4	4.75
Body Mass Index	14	2	10	2	8	8	3	3	6.25
Could not see doctor due to cost	5	6	12	4	6	6	6	6	6.375
Flu shot/spray past 12 months	8	9	2	5	12	12	7	7	7.75
Age group	3	13	11	7	3	3	13	13	8.25
Education	11	11	8	8	9	9	8	8	9
Race	7	14	9	13	1	1	14	14	9.125
Diabetes	12	7	6	10	10	10	10	10	9.38
Smoker Status	10	8	4	11	14	14	9	9	9.875
Smoked at least 100 cigarettes	13	10	13	12	11	11	11	11	11.5
Veteran Status	9	12	14	14	13	13	12	12	12.375

*Indicates the level of importance with 1 meaning the most important variable.

Since logistic regression model is identified as one of the best-performing models in this study, we select it for further exploration into the risk factors. Another advantage that logistic regression offers is the meaningful interpretation of odds ratios that are computed for each level for predictors. Table 8 presents the weighted odd ratios (OR) and their corresponding 95% CI obtained using multiple logistic regression model on the entire dataset.

**Table 8:** Summary information from logistic regression model on the entire dataset.

Predictors	Odds ratio	95% CI for Odds ratio
**Chronic Obstructive Pulmonary Disease**		
Yes (Ref.) *		
No	0.209	(0.162, 0.270)
**Income**		
Below $25000 (Ref.)		
$25000–$50000	0.836	(0.631, 1.108)
$50000 or more	0.668	(0.518, 0.861)
Don’t know/Not Sure	0.654	(0.491, 0.871)
**Sex**		
Male (Ref.)		
Female	1.535	(1.255, 1.879)
**Employment**		
Employed for wages (Ref.)		
Out of work	0.983	(0.645, 1.498)
Homemaker	1.198	(0.732, 1.961)
Student	0.808	(0.473, 1.380)
Retired	1.018	(0.743, 1.394)
Unable to work	1.332	(0.950, 1.868)
**Body Mass Index**		
Underweight (Ref.)		
Normal Weight	0.815	(0.412, 1.613)
Overweight	0.916	(0.467, 1.795)
Obese	1.266	(0.648, 2.475)
**Could not see doctor due to cost**		
Yes (Ref.)		
No	0.770	(0.599, 0.990)
**Flu shot/spray past 12 months**		
Yes (Ref.)		
No	0.729	(0.603, 0.881)
**Age group**		
18–24 (Ref.)		
25–34	0.696	(0.456, 1.062)
35–44	0.714	(0.467, 1.092)
45–54	0.587	(0.385, 0.894)
55–64	0.472	(0.310, 0.719)
65 and above	0.304	(0.191, 0.485)
**Education**		
Below High School (Ref.)		
High School	0.696	(0.473, 1.025)
Some college or more	0.834	(0.577, 1.207)
**Race**		
White, Non-Hispanic (Ref.)		
Black, Non-Hispanic	1.365	(1.029, 1.811)
Asian, Non-Hispanic	0.697	(0.237, 2.050)
Hispanic	0.732	(0.455, 1.179)
Other	1.058	(0.667, 1.677)
**Diabetes**		
Yes (Ref.)		
No	0.717	(0.562, 0.915)
Pre-diabetes or borderline diabetes	0.898	(0.537, 1.502)
**Smoker Status**		
Current smoker (Ref.)		
Former smoker	0.965	(0.734, 1.268)
Never smoked	1.500	(0.449, 5.006)
**Smoked at least 100 cigarettes**		
Yes (Ref.)		
No	0.687	(0.209, 2.258)
**Veteran Status**		
Yes (Ref.)		
No	1.254	(0.899, 1.749)

*(Ref.) indicates Reference Group

The relationship between asthma and various predictors used in the study can be examined from the results in Table 8. The insights of the predictors which show statistically significant odds ratios are discussed here in brief. People without COPD were about 80% less likely to report asthma compared to people with COPD [OR = 0.209, 95% CI (0.162, 0.270)]. Compared to people in the lowest income level, those with an income $50000 or more were about 33% less likely [OR = 0.668, 95% CI (0.518, 0.861)] and those who did not know or were not sure about their income were about 35% less likely [OR = 0.654, 95% CI (0.491, 0.871)] to report asthma. Regarding sex, females were 1.535 times more likely [OR = 1.535, 95% CI (1.255, 1.879)] to have reported asthma compared to males.

The participants’ likelihood of reporting asthma was also influenced by their access to health care. Participants who had no financial barriers to seeing a doctor when needed were 23% less likely [OR = 0.770, 95% CI (0.599, 0.990)] to report asthma than those who did have financial barriers. Contrary to expectations, people who did not take flu shot/spray in the past 12 months were about 27% less likely [OR = 0.729, 95% CI (0.603, 0.881)] to have reported asthma compared to those who did take flu shot/spray in the past 12 months.

The odds of self-reported asthma were less among people in higher age groups. Compared to people in 18–24 years age group, those in the 45–54, 55–64, and 65 and above years group were about 41% [OR = 0.587, 95% CI (0.385, 0.894)], 53% [OR = 0.472, 95% CI (0.310, 0.719)], and 70% [OR = 0.304, 95% CI (0.191, 0.485)] less likely to report asthma, respectively. Regarding race, Black, non-Hispanic were found 1.365 times more likely [OR = 1.365, 95% CI (1.029, 1.811)] to have reported asthma compared to White, non-Hispanic. Diabetes was also found to have an association with self-reported asthma as people without diabetes were about 28% less likely [OR = 0.717, 95% CI (0.562, 0.915)] to report asthma than people with diabetes. Although various other relationships between the predictors and asthma have been quantified in terms of odds ratio in Table 8, those were not statistically significant.

## Discussion

To the best of our knowledge, this is the first study that attempted to predict asthma using modern machine learning models based on demographic/socioeconomic, personal habits, and health characteristics collected from a national survey.

One of the objectives of this study was to assess and compare the performance of machine learning models in predicting asthma among Michigan adults using an imbalanced training dataset from the 2019 BRFSS. The modern machine learning techniques require the assumption of approximately balanced majority and minority classes. In this regard, the imbalanced dataset issue was addressed using two popular synthetic data generation methods, namely ROSE and SMOTE. These methods were employed to oversample the minority class and under sample the majority class to have an approximately balanced training dataset. We observed that having a balanced training set improves model performance when evaluated on the testing set. For the ROSE-adjusted models, this was demonstrated by about 25% increase in the AUC value, in the best case, with partial least squares technique. In our study, we found that the ROSE method had advantages over SMOTE based on metrics that are relevant to studying model performances in the presence of imbalanced classes. Previous studies also established improvements in model performance by use of resampling techniques. Study in [12] showed an 11% increment in AUC among models developed for sleep/wake prediction from Fitbit data. Another study reported improved performances of random forest and naïve bayes, among others, for predicting incident diabetes in a dataset that was balanced using SMOTE technique [11]. Therefore, our study further demonstrates the improved potentialities of machine learning models coupled with imbalanced data modeling techniques.

Along with the performance metrics, this study also reported the importance of predictors for making predictions in each of the models trained using ROSE method. As shown in Table 7, COPD was identified as the most important predictor of asthma, and this is not surprising. Although these two diseases are different, they have various similar symptoms thus, are highly associated with each other [33]. Other important predictors that contributed to predictions in most of the models were income, sex, and employment. Somewhat surprising is that none of the smoking-related predictors was highly important in any of the models examined. This finding contrasts with prior studies conducted on the US population, which have revealed a significant association between smoking and asthma [6–8]. To some extent, this could be due to predictors being highly correlated, and thus the effects of smoking-related variables may have been explained by other predictors.

This study also examined the risk factors of diabetes by exploring the best-performing model in further detail. Choosing logistic regression as one of the most successful models based on performance measures, we found some anticipated results in line to previous studies as well as other interesting relationships. Table 8 indicates that people with COPD were more likely to report asthma than people without COPD, which was an expected finding. Regarding income levels, we found that higher income leads to lower odds of self-reported asthma. Previous studies have reported the association between income and asthma in a similar line [6, 8]. Literature that analyzed previous BRFSS datasets demonstrated higher likelihood of reporting asthma by females [6, 8], and this study further validates this finding. This ongoing disparity might indicate either the absence or ineffectiveness of intervention programs for asthma control among female population.

Another risk factor identified by the study is the inability to see doctor due to the cost involved. This demands the need to make health care access affordable to people with financial struggles. Interestingly, the study found that people who did not take flu shot/spray in the past 12 months were less likely to report asthma. This association could be the other way too: adults who had asthma could be more likely to show up for the flu shot. People in all other higher age-groups were less likely to report asthma than people in 18–24 years age group. Again, study in [6] found that adults aged 35–64 and ≥65 years were less likely to report current asthma than adults aged 18–34 years. Regarding race, once again in line to previous findings [7, 8], Black, non-Hispanic population had increased odds of self-reported asthma. The American Lung Association also reports the same regarding asthma disparities among races [34]. Finally, our study found that people without diabetes have lower odds of reporting asthma. A similar association between asthma and diabetes was reported in separate studies among the US [15] and Korean populations [17].

Our study is not without limitations. Firstly, BRFSS relies solely on self-reported data and, thus, may be subject to recall bias and errors. Secondly, our selection of predictors was limited to the literature reviewed, so we could not comprehensively incorporate every potential predictor of asthma, particularly those pertaining to clinical characteristics as are measured in other surveys such as NHANES. Thirdly, it is important to note that no inferences about causality of observed associations can be made due to the cross-sectional sampling design of BRFSS. Fourthly, regarding the use of ROSE method, it is reported that random under sampling can potentially remove certain important examples, and random oversampling can lead to overfitting [35]. Also, for large datasets, oversampling can be computationally intensive. Finally, although we incorporated CDC-computed individual respondent weights for association tests and logistic regression analyses for exploration of risk factors, the existing R packages for machine learning did not consider weights in the modeling process. Due to this reason, none of the machine learning models in this study considered survey weights. A recent paper in [36] discusses the implications of including survey weights in machine learning models and we plan to carry out a future study in that direction.

## Conclusion

The straightforward application of the predictive modeling techniques based on the imbalanced training data indicated poor performances of those techniques. We were able to demonstrate improved performance of modern machine learning algorithms when trained on a sample balanced using synthetic data generation techniques. The investigation into asthma risk factors produced evidence in line with previous studies. We believe that findings could guide early screening of at-risk asthma patients. Also, this study may serve as a literature for building more advanced predictive models to predict asthma in the future. Apart from building predictive models and identifying risk factors, the implementation of early intervention for people in higher-risk groups is crucial for improving public health.
